# Efficient biosynthesis of D/L-alanine in the recombinant *Escherichia coli* BL21(DE3) by biobrick approach

**DOI:** 10.3389/fbioe.2024.1421167

**Published:** 2024-08-12

**Authors:** Muhammad Naeem, Shimiao Hao, Mengqiu Chu, Xuan Zhang, Xinyan Huang, Jiaying Wang, Guangzheng He, Baohua Zhao, Jiansong Ju

**Affiliations:** ^1^ College of Life Science, Hebei Normal University, Shijiazhuang, China; ^2^ Hebei Collaborative Innovation Center for Eco-Environment, Shijiazhuang, China

**Keywords:** D/L-alanine, biobrick method, gene expression, D-glucose, optimization, SDS-PAGE, whole cell catalysis

## Abstract

Alanine is the most abundant chiral amino acid that exists into the D-alanine or L-alanine forms with diverse applications in the biomedical, pharmaceutical, plastics, and food industries. D/L-alanine production can be carried out through chemical, microbial fermentation, and biocatalytic methods and not much effective due to complicated processes or purification issues and is still challenging to achieve a higher yield. In the present study, biobrick method was utilized for efficient production of D/L-alanine in the recombinant *Escherichia coli* BL21(DE3) with tandem three-gene co-expression plasmid. Firstly, the co-expression plasmid pET-22bNS-DadX-Ald-Gdh containing three genes, alanine dehydrogenase (*ald)*, alanine racemase (*dadX)*, and glucose dehydrogenase (*gdh)* from *Bacillus pseudofirmus* OF4 were successfully constructed and introduced into the *E. coli* BL21(DE3) strain. Then, under optimized conditions in the whole-cell biocatalytic reaction [20 mM Na_2_CO_3_-NaHCO_3_ (pH 10.1), 200 mM D-glucose, 200 mM sodium pyruvate, and 200 mM ammonium chloride], the concentration of D-alanine and L-alanine reached the maximum value (6.48 g/L and 7.05 g/L) after 3.0 h reaction time at 37°C under 180 rpm rotation. Meanwhile, promoter replacement experiments and Western blot analysis revealed that the expression level of protein OF4Ald had a significant effect on the production of D/L-alanine, indicating that alanine dehydrogenase might be the rate-limiting enzyme for D/L-alanine synthesis. This study provides a simple, feasible, and efficient biosynthesis process of D/L-alanine, which could explore emerging applications for large-scale production of industrial bioproducts.

## Graphical Abstract

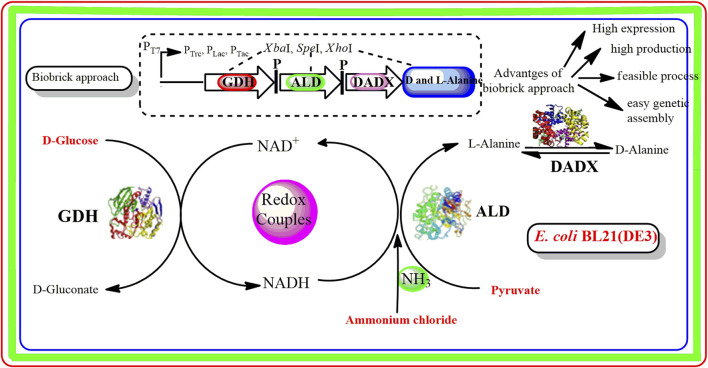

## 1 Introduction

Alanine is the most abundant chiral amino acid and acts as a central pool in protein metabolism (Al Mughram et al., 2023). The molecular structure and chiral nature of alanine revealed that it exists in the D-alanine or L-alanine forms (Moozeh et al., 2015). D/L-alanine has different applications in the pharmaceutical, plastics, and food industries (Xin et al., 2022). In pharmaceutical industries, it is used for the preparation and formulation of novel *β*-lactam antibiotics. In food industries, it is added as artificial sweeteners to enhance the quality of food products. In plastic industries to reduce the risk of environmental pollution, they are utilized as feedstocks for the engineering of biodegradable thermoplastics such as polyester amides (PEAs), polylactic acid (PLA), and polyester imides (PEIs) (Sharma et al., 2021). Due to these emerging applications in different industrial sectors, D/L-alanine production has reached annual demand with >50,000 tons in the global market (Liu et al., 2022).

Ald, Gdh, and DadX have different roles and biological importance in many microorganisms. Alanine dehydrogenase (Ald, EC 1.4.1.1) belongs to the amino acid dehydrogenase that catalyzes the conversion of pyruvate into L-alanine (Dave and Kadeppagari, 2019). The *ald* gene encoded the function of alanine dehydrogenase and has been purified from many microorganisms such as *Bacillus subtilis*, *Rhodobacter capsulatus*, *Thermotoga maritima*, and *Bacillus cereus* (Gu et al., 2023). It has different physiological roles in some microorganisms. In *T. maritima*, it plays an important role in the formation of the peptidoglycan layer and stabilizes the structural components of the cell wall (Miyamoto, 2024). It is involved in the growth and sporulation of *B. subtilis* because it produces the pyruvate through the oxidative deamination. The pyruvate then generates energy for sporulation through the tricarboxylic acid cycle. It also plays an important role in the nitrogen cycle through the nitrogen fixation process and enhances the efficiency of biomass accumulation (Dave and Kadeppagari, 2019). Glucose dehydrogenase (Gdh, EC 1.1.1.118) catalyzed the oxidation of glucose into gluconolactone through NADH regeneration from NAD^+^ (Yan et al., 2022). The *gdh* gene is required for the activity of glucose dehydrogenase and plays an important role in the biosynthetic pathway of D/L-alanine. It has been purified from many microorganisms such as *Pseudomonas moorei*, *B. subtilis*, and *Corynebacterium glutamicum* (Wu Y. et al., 2022; Zou et al., 2024). It plays a significant role in the phosphate solubilization through acidification that occurs in the periplasmic space of bacteria (Wu X. et al., 2022). It catalyzed the oxidation of glucose into the formation of gluconic acid and 2-ketogluconic acid contributed to the acidification process. This pathway is particularly important for some enteric bacteria to survive in the low-phosphate and aquatic environments [4]. It also plays an important role in the formulation of novel β-lactam antibiotics such as penicillin and the production of biofuels such as bioethanol (Yan et al., 2022).

Alanine racemase (DadX, EC 5.1.1.1) catalyzed the racemization reaction by converting the L-alanine into D-alanine (Azam and Jayaram, 2016). The *dadX* gene encoded the function of alanine racemase and has been purified from many microorganisms such as *Clostridium perfringens* and *Bacillus pseudofirmus* (Dong et al., 2018; Israr et al., 2019). It plays a significant role in the production of antibacterial drugs. For example, d-Cycloserine is a novel inhibitor of alanine racemase used for pulmonary tuberculosis. It inhibited the activity of alanine racemase and blocked the production of d-alanine which is utilized by bacteria for the formation of cell wall (Azam and Jayaram, 2016).

D/L-alanine synthesis can be carried out through chemical, fermentation, and biocatalytic methods (Fan et al., 2021). Chemically, D/L-alanine is synthesized by heating the solution of ammonium ions and glyceraldehyde at 22°C and produces only a 4.5% yield of alanine (Weber, 1985). The yield obtained through the chemical method is very low and has no practical application due to toxicity issues and high costs. Previous literature reported that some microbial strains were engineered for D/L-alanine production through fed-batch fermentation. For example, *Arthrobacter oxydans* HAP-1 strain produced the maximum D/L-alanine of about 75.6 g/L with 120 g/L glucose consumption in 120 h through fed batch fermentation (Hashimoto and Katsumata, 1998). Using the fed-batch mode, 88 g/L of D/L-alanine was obtained by *E. coli* ALS929 through microbial fermentation in 48 h (Smith et al., 2006). These studies revealed several drawbacks to the fermentation process, such as the release of byproducts in the fermentation broth that hindered the purification process and ultimately produced a low yield of D/L-alanine (Li et al., 2018). However, the yield of D/L-alanine through biocatalytic synthesis is still low, and expensive substrates are utilized for optimizing process that are commercially unavailable for large-scale production (Li et al., 2018). Different efforts were also made for efficient D/L-alanine production through coupling methods with combinations of different enzymes or biocatalysts, including the format dehydrogenase (FDH), D-amino acid aminotransferase (DAAT), alanine dehydrogenase (Ald) and alanine racemase (Alr) (Galkin et al., 1997a). The coupling reaction was carried out by heating the ammonium format with alpha-keto acid at 37°C for 10 h, which produced only an 80% yield of D/L-alanine (Galkin et al., 1997b). The yield obtained through this method is still low due to some disadvantages, such as additional gene cloning of the recombinant plasmids being required due to degradation of the coenzymes. Therefore, the above methods are not suitable for efficient production of D/L-alanine. To overcome these limitations, a simple and reliable alternative method is urgently needed for the high-scale synthesis of D/L-alanine.

Biobrick approach has emerged as a simple and efficient method that reveals the expression level of different genes in several biosynthetic pathways (Matsumura, 2020). Through this approach, different gene fragments are digested with isocaudomers restriction endonucleases (*Nde*I, *Xho*I, *Not*I, *Spe*I, etc.). As a result, short DNA fragments (biobricks) are generated that can efficiently ligated into the living cells such as *E. coli* for the construction of a new biological system (Yamazaki et al., 2017). Biobrick approach has several advantages over conventional methods. Firstly, through biobrick approach, it becomes easier to design and assemble the larger assembly units by developing new genetic vectors. On the other hand, the design of traditional cloning methods is much more complicated for the manipulation of larger genetic circuits (Braman and Sheffield, 2019). In contrast to traditional methods, several genes can be assembled by biobrick method into the recombinant expression plasmid rather than single gene transfer thereby facilitating the high expression level of several genes. Biobrick methods are cost-effective than traditional methods as there is no need for additional cloning which reduces the experimental costs (Chaudhari and Hanson, 2021). Overall, these features provide an excellent opportunity for exploring the efficient production of D/L-alanine. However, screening of some functional genes for high D/L-alanine production through the biobrick method is still lacking and further needs to study.

In this study, biobrick method was utilized for efficient D/L-alanine production in the recombinant *E. coli* BL21(DE3) strain harboring the co-expression plasmid containing *ald*, *dadX*, and *gdh* genes derived from *B. pseudofirmus* OF4. To achieve the maximum conversion rate of D/L-alanine synthesis, different conditions were optimized in the whole-cell catalytic reaction. The synthetic efficiency of different recombinant strains was evaluated through the replacement of the T7 promoter of the pET-22b(+) expression vector with *P*_*Lac*_, *P*_*Tac*_, and *P*_*Trc*_ promoters. Furthermore, the relationship between gene expression and different promoters was explored by Western blot analysis.

## 2 Materials and methods

### 2.1 Plasmids, strains, and reagents

*Escherichia coli* DH12S and BL21(DE3) were used for DNA molecular cloning and recombinant protein expression. Plasmids pUC57-Lac, pUC57-Tac, and pUC57-Trc containing gene fragments of different promoters P_Lac_, P_Tac_, and P_Trc_ were obtained from Sangon-Biotech (Shanghai, China). The expression vector pET-22b(+) was used for protein expression (Novagen, Darmstadt, Germany). Restriction enzymes (*Nde*I, *Xho*I, *Not*I, *Spe*I, *Nhe*I, *Bgl*II), T4 DNA ligase, and ExTaq DNA polymerase were purchased from Takara (Dalian, China). Horseradish peroxidase (HRP)-labeled goat anti-mouse IgG was purchased from Solarbio (Beijing, China). Ampicillin, pyridoxal 5′-phosphate (PLP), NAD^+^, IPTG, and sodium pyruvate were purchased from Sangon-Biotech (Shanghai, China). D/L-Alanine was obtained from Sigma-Aldrich (St. Louis, MO, United States). Fifteen male mice were purchased from Hebei Medical University, China. All other chemicals and reagents used in this study were analytical grade.

### 2.2 Construction of tandem three-gene expression plasmids

For the construction of the co-expression vector, the recognition site of *Spe*I (ACTAGT) was introduced into the plasmid pET-22b (+) by site-directed mutagenesis using primer pairs of Spe-F01 and Spe-R01 to generate the pET-22bS vector (Table 1). The cloning region of pET-22bS was amplified by PCR reaction using the primer pairs of BgNhe-F01 [Forward primer, containing recognition site of Isoschizomer *Nhe*I (GCTAGC)] and Spe-R01 (Reverse primer). The PCR products were digested by restriction enzymes *Bgl*II and *Spe*I and inserted into the same restriction sites of vector pET-22bS to generate a new vector named pET-22bNS with recognition sites of *Spe*I and *Nhe*I.

**TABLE 1:** Shows the different primers used in this study.

Primer	Target sequence (5′–3′)	Description
Spe-F01	AGG​AGG​AACT​AGTTCC​GGA​TTG​GC	pET-22bS
Spe-R01	GCC​AAT​CCG​GAA​CTA​GTT​CCT​CCT	
BgNhe-F01	GAGA​TCTCGA​TGCT​AGCAAA​TTA​ATA​CGA​CTC	*Bgl*II and *Nhe*I
DadX-F01	CAT​ATGAAG​ACG​AGC​AGT​TTT​AGA	*dadX* gene cloning
DadX-R01	CTC​GAGGTT​CTC​TTC​GTA​ATA​TCT​CGG​AAC	
Ald-F01	CAC​GCAT​ATGATT​ATC​GGT​ATT​CCA	*ald* gene cloning
Ald-R01	AGCCTC​GAGTGC​TTG​AAC​AGG​TGT​TTT​C	
Gdh-F01	GCAT​ATGAAA​AGA​CTT​ATA​GCA​GT	*gdh* gene cloning
Gdh-R01	AGCG​GCCGCT​TCA​CTT​CTA​ATC​AAT​TC	

^a^ The underlined nucleotide sequences are targeted sites for *Bgl*II, *Nhe*I, *Spe*I, *Nde*I, *Xho*Ⅰ, *and Not*I restriction endonucleases.

Three genes *ald*_OF4_, *dadX*_OF4_, and *gdh*_OF4_ were amplified by PCR reaction from the genomic DNA of *B. pseudofirmus* OF4 (GenBank ID: ADC50010.1, ADC50009.1, ADC51909.1) using primer pairs listed in Table 1 and cloned into plasmid pET-22bNS using the *Nde*I and *Xho*I or *Not*I restriction sites to construct the expression plasmids pET-22bNS-DadX, pET-22bNS-Ald and pET-22bNS-Gdh, respectively. Then, plasmid pET-22bNS-DadX was digested with *Bgl*II and *Spe*I, and cloned into the *Bgl*II and *Nhe*I (Isocaudomer of *Spe*I) sites of plasmid pET-22bNS-Ald, generating the recombinant tandem plasmid pET-22bNS-DadX-Ald. Finally, plasmid pET-22bNS-Gdh was digested with *Bgl*II and *Spe*I and cloned into the *Bgl*II and *Nhe*I sites of plasmid pET-22bNS-DadX-Ald to construct the tandem three-gene co-expression plasmid pET-22bNS-DadX-Ald-Gdh through biobrick approach.

Plasmids pUC57-Lac, pUC57-Tac, and pUC57-Trc were digested with restriction enzymes *Bgl*II and *Xho*I, and P_T7_ promoter of plasmid pET-22bNS was replaced with different promoters (P_Lac_, P_Tac_, and P_Trc_) to construct the expression vectors pET-22bNS_Lac_, pET-22bNS_Tac_, and pET-22bNS_Trc_. Three genes, *ald*_*OF4*_, *dadX*_*OF4*_, and *gdh*_*OF4*_ were then cloned into vectors pET-22bNS_Lac_, pET-22bNS_Tac_, and pET-22bNS_Trc_, separately, and different combinations of tandem three-gene co-expression plasmids with different promoters were successfully constructed.

### 2.3 Heterogeneous protein expression and purification

The recombinant plasmids pET-22bNS-Ald, pET-22bNS-DadX, pET-22bNS-Gdh, and tandem three-gene co-expression plasmid pET-22bNS-Ald-DadX-Gdh were transformed into the *E. coli* BL21(DE3) strain by chemical transformation. The strains were cultured into 100 mL of LB medium containing ampicillin (100 mg/mL) at 37°C and placed in the rotary shaker at 180 rpm for 6 h until the optical density (OD_600_) reached around 0.5–0.6. Then, protein expression was induced with supplementation of 0.5 mM isopropyl thio-*β*-D-galactoside (IPTG), and growth of cells was continuously progressed at 30°C for 15 h. Recombinant cells were collected through centrifugation at 8,000 rpm for 10 min, and cells were disrupted by ultra-sonication into the 10 mL lysis buffer containing 50 mM NaH_2_PO_4_ (pH 8.0), 300 mM NaCl, and 10 mM imidazole. The cell lysis was again centrifugated at 10,000 rpm for 15 min to discard the precipitates, and recombinant proteins with His_6_-tagged were purified by Ni-NTA super-flow resin (Qiagen, Valencia, CA, United States) according to the manufacture instructions.

The purified proteins OF4Ald, OF4DadX, and OF4Gdh were dialyzed and desalted at 4°C against the phosphate buffer (20 mM, pH 8.0) containing the 0.5 mM of EDTA, 0.01% of 2-mercaptoethanol, and appropriate coenzyme (PLP or NAD^+^). The purity of recombinant proteins was examined by SDS-PAGE analysis.

### 2.4 Protein expression level detection

The experiment was carried out on fifteen male mice and divided equally into three groups (five mice in each group). Each group was immunized four times via peritoneal injections (50, 100, 150, and 200 μg) containing the mixture of recombinant proteins OF4DadX, OF4Ald, and OF4Gdh. The serum was collected and centrifuged at 2,000 rpm for 20 min. The expression level of OF4DadX, OF4Ald, and OF4Gdh proteins was analyzed by Western blotting analysis using antibodies against purified proteins OF4DadX, OF4Ald, and OF4Gdh from *B. pseudofirmus* OF4 and detected using the high sensitivity chemiluminescence kit (Kangwei, Beijing) according to the manufacturer’s manual and expressed in percentage.

### 2.5 D/L-alanine detection

D-alanine content was detected by the D-amino acid oxidase method as described previously (Ju et al., 2005; Ju et al., 2009). Following the standard protocols, the reaction mixture was prepared by dissolving the 200 mM of Tris-HCl, pH 8.0, 0.1 mg/mL of 4-aminoantipyrine, 0.1 mg/mL of *N*-ethyl-*N*-(2-hydroxy-3-sulfopropyl)-3-methylaniline sodium salt (TOOS), 2 units of peroxidase, and 0.1 unit of D-amino acid oxidase (pkDAAO, Sigma) to make the final volume of 200 μL. The reaction was carried out at 37°C for 20 min, and absorbance was measured at 550 nm by an Epoch Microplate Spectrophotometer (BioTek, United States). Finally, the amount of D-alanine in the reaction mixture was calculated by calibrating the standard curve.

Products of the reaction mixture containing D-alanine and L-alanine were detected through HPLC analysis (Ju et al., 2009). In this method, D/L-alanine in the reaction mixture was derivatized at 25°C for 2 min with 280 mM borate buffer (pH 9.0), containing 0.2% of *o*-phthaldialdehyde (OPA) (Sigma, Germany) and 0.2% of *N*-*tert*-butyloxycarbonyl-L-cysteine (Boc-L-Cys) (Fluka, Switzerland). Then, 15 μL of the reaction mixture sample was loaded into the Nova-PACK C18 column (4 μm, 3.9 × 300 mm; Waters, Milford, MA, United States), and the reaction products of D/L-alanine were quantitatively estimated by a Shimadzu RF-20A Prominence Fluorescence Detector and expressed in millimolar units (Kyoto, Japan). Moreover, the amount of sodium pyruvate was also determined by the HPLC method, as described previously with some modifications (Ewaschuk et al., 2004). In this method, 10 μL of the reaction mixture was loaded into a WondaSil C18-WR column (5.0 μm, 4.6 × 250 mm), and the amount of sodium pyruvate was detected at 30°C and 210 nm wavelength with aqueous 0.02 mM KH_2_PO_4_ (pH 2.4) as the mobile phase. Finally, the amount of sodium pyruvate was estimated by a Shimadzu RF-20A Prominence Fluorescence Detector and expressed in percentage (Kyoto, Japan).

### 2.6 Optimizing conditions for D/L-Alanine synthesis

Different reaction conditions were optimized to achieve the maximum conversion rate for D/L-alanine synthesis using the following parameters.

#### 2.6.1 Concentration of inducer

*Escherichia coli* BL21(DE3) strain harboring the tandem three-gene co-expression plasmids pET-22bNS-Ald-DadX-Gdh, and pET-22bNS-Ald_Trc/Tac/Lac_-DadX_Trc/Tac/Lac_-Gdh_Trc/Tac/Lac_ were inoculated into 100 mL LB medium with ampicillin (100 mg/mL) and incubated at 37°C in a rotary shaker at 180 rpm for 1.5 h. When the optical density (OD_600_) reached 0.5–0.6, IPTG with different concentrations (0, 0.2, 0.4, 0.6, 0.8, and 1.0 mM) was added into the culture, and the growth was continued at 30°C for 15 h.

The bacterial pellets were collected and rinsed twice with 50 mM NaH_2_PO_4_ buffer (pH 8.0) and 0.33 g of the harvested cells were resuspended into 10 mL reaction solution (1/30 g/mL) [20 mM Na_2_CO_3_-NaHCO_3_ (pH 10.1) with 50 mM sodium pyruvate, 50 mM ammonium chloride, 50 mM D-glucose, 10 μM PLP, and 0.5 mM NAD^+^] and incubated at 37°C for 1.5 h under 180 rpm rotation. After centrifugation, the optimal concentration of IPTG was determined by detecting the amount of D-alanine in the reaction solution (cell-free) by the D-amino acid oxidase (DAAO) method.

#### 2.6.2 Concentration of coenzymes

For determination of NAD^+^ concentration, 0.33 g of the harvested cells were resuspended into 10 mL Na_2_CO_3_-NaHCO_3_ buffer (20 mM, pH 10.1) containing the 50 mM of sodium pyruvate, 50 mM of ammonium chloride, 50 mM of D-glucose substrates, 10 μM of PLP and varying concentrations of NAD^+^ (0 mM, 0.1 mM, 0.2 mM, 0.3 mM, 0.4 mM, 0.5 mM), and reaction mixture was incubated at 37°C for 1.5 h under 180 rpm rotation. The optimal concentration of NAD^+^ was determined by analyzing the amount of D-alanine in the reaction solution.

For determination of NADP^+^ and PLP coenzymes concentration, 0.33 g of the harvested cells were resuspended into the same reaction solution with substrates containing various combinations of exogenous coenzyme [NADP^+^ (0 or 0.4 mM) with PLP (10 μM) or PLP (0 or 10 μM) without NAD^+^ and NADP^+^], then incubated at 37°C with rotation at 180 rpm for 1.5 h. The optimum concentrations of NADP^+^ and PLP were evaluated according to the D-alanine content of the reaction solution.

#### 2.6.3 Reaction temperature

For determination of optimal reaction temperature, 0.33 g of the harvested cells were resuspended into 10 mL Na_2_CO_3_-NaHCO_3_ buffer (20 mM, pH 10.1) containing 50 mM of sodium pyruvate, 50 of mM ammonium chloride, 50 of mM D-glucose substrates, then incubated at various temperatures (31°C, 34°C, 37°C, 40°C, 43°C, 46°C) with 180 rpm rotation for 1.5 h. After centrifugation, the optimum reaction temperature was determined by analyzing the amount of D-alanine in the reaction solution.

#### 2.6.4 pH value

In case of optimal pH value determination, 0.33 g of the harvested cells were resuspended into 10 mL of Na_2_CO_3_-NaHCO_3_ buffer (20 mM, different pH values: 9.2, 9.5, 9.8, 10.1, 10.5, 10.8) containing the 50 mM of sodium pyruvate, 50 mM of ammonium chloride, 50 mM of D-glucose substrates, and was kept at 37°C with 180 rpm rotation for 1.5 h. After centrifugation, the optimal pH value of the buffer was recorded according to the D-alanine content of the reaction solution.

#### 2.6.5 Concentration of substrates

The 0.33 g harvested cells were resuspended into 10 mL of Na_2_CO_3_-NaHCO_3_ buffer (20 mM, pH 10.1), and varying concentrations of three substrates (sodium pyruvate/ammonium chloride/D-glucose: 50, 100, 150, 200, 250, 300, 350, 400 mM), followed by incubation at 37°C with agitation at 180 rpm for 1.5 h. Subsequently, the concentration of the substrates was optimized by analyzing the amount of D-alanine in the reaction solution.

#### 2.6.6 Mass-to-volume ratio of cell pellets

The cell pellets (wet weight) at various mass-to-volume ratios (1/5, 1/10, 1/15, 1/20, 1/30, and 1/40 g/mL) were separately resuspended into 10 mL of Na_2_CO_3_-NaHCO_3_ buffer (20 mM, pH 10.1) containing the 200 mM of sodium pyruvate, 200 mM of ammonium chloride, 200 mM of D-glucose substrates, then incubated at 37°C shaking at 180 rpm for 1.5 h. After the removal of bacterial cells by centrifugation, the optimum mass-to-volume ratio of cell pellets was screened out by detecting the D-alanine content of the reaction solution.

#### 2.6.7 Concentration of surfactants

For determination of surfactants effect on D/L-alanine synthesis, the 10 mL of optimal reaction solution containing [Na_2_CO_3_-NaHCO_3_ (20 mM, pH 10.1), 200 mM of sodium pyruvate, 200 mM of ammonium chloride, 200 mM of D-glucose] and varying concentration of surfactants [CTAB (0.0, 0.1, 0.2, 0.3, 0.4, 0.5 mg/mL), or Triton X-100 (0.0, 0.5, 1.0, 1.5, 2.0, 2.5%), CHAPS (0.0, 0.1, 0.2, 0.3, 0.4, 0.5 mg/mL), SDS (0.0, 0.1, 0.2, 0.3, 0.4, 0.5 mg/mL), separately] was dissolved into the 0.5 g of the cell pellets. The reaction mixture was mixed well and incubated at 37°C and 180 rpm for 1.5 h. After centrifugation, the optimum concentration of surfactants was explored by analyzing the D-alanine content.

#### 2.6.8 Reaction time

For determination of the optimal reaction, the 10 mL of optimal reaction solution [20 mM Na_2_CO_3_-NaHCO_3_ (pH 10.1), 200 mM of sodium pyruvate, 200 mM of ammonium chloride, 200 mM of D-glucose] without any surfactants mixed well with 0.5 g of cell pellets and incubated at 37°C with shaking at 180 rpm for varying reaction times (0.5, 1.0, 1.5, 2.0, 2.5 3.0, 3.5, 4.0, 4.5, 5.0, 5.5, and 6.0 h). The optimal reaction time was recorded by analyzing the D-alanine content in the reaction solution.

#### 2.6.9 Reusability of bacterial pellets

The reaction mixture containing the 10 mL optimal reaction solution was incubated at 37°C at 180 rpm for 3.0 h through resuspension of 0.5 g of the cell pellets. After centrifugation, the collected bacterial pellets were resuspended in a fresh reaction solution (10 mL, 1/20 g/mL) as the catalysts for the next round of D/L-alanine synthesis. The reusability of bacterial pellets was recorded according to the amount of D-alanine in the supernatant.

### 2.7 Effect of promoter

The bacterial strains harboring all 21 tandem co-expression plasmids were pre-cultured and induced at 30°C for 15 h without induction with IPTG, respectively. Then, 10 mL of optimal reaction solution was mixed well with 0.5 g of cell pellets at 37°C for 3.0 h under 180 rpm rotation. After centrifugation, the effect of the promoter was determined by analyzing the D-alanine content in the reaction solution.

### 2.8 Sequencing analysis

The nucleotide sequences of all constructed recombinant plasmids were confirmed by an Illumina NovaSeq 6000 platform (Illumina, United States). The nucleotide and amino acid sequences of proteins OF4DadX, OF4Ald, and OF4Gdh were available on the GenBank website according to the accession numbers ADC50009.1, ADC50010.1, and ADC51909.1.

## 3 Results

### 3.1 Construction of tandem three-gene co-expression vectors

**FIGURE 1:**
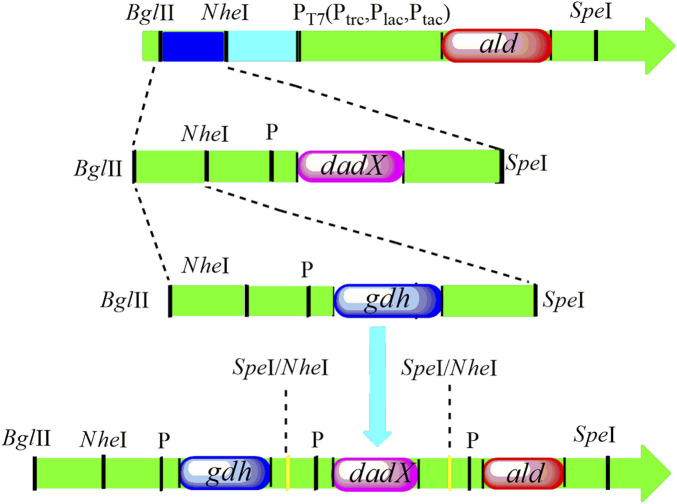
The construction design of tandem three genes with replacement of different promoters P_T7_ (P_Lac_, P_Tac_, and P_Trc_) following the biobrick approach. The recognition site of *Spe*I was inserted into the upstream part of the *ald* gene. The restriction endonucleases *BglII* and *SpeI* facilitate the double digestion of the *dadX* gene and cloned into the *ald* gene. Then, *gdh* gene was cleaved with *Bgl*II and *Spe*I, and positioned into the *Bgl*II and *Nhe*I sites of *ald-dadX* gene. Finally, through the action of *Nhe*I and *Spe*I (Isocaudamers), the tandem three gene *ald-gdh-dadX* with different promoters was constructed.

Three genes *ald*_OF4_, *dadX*_OF4_, and *gdh*_OF4_ were amplified from *B. pseudofirmus* OF4 and cloned into the expression vector pET-22bNS to construct the plasmids pET-22bNS-Ald, pET-22bNS-DadX, pET-22bNS-Gdh and tandem three-gene co-expression plasmid pET-22bNS-Ald-DadX-Gdh (Figure 1). Furthermore, 21 tandem three-gene co-expression plasmids with different promoters (P_Lac_, P_Tac_, and P_Trc_) combinations were constructed through the biobrick approach. The constructed recombinant plasmids were then introduced into the *E. coli* BL21(DE3) strain by chemical transformation. The successful gene expression of each recombinant plasmid under the regulation of different promoters ensured the high synthetic efficiency of D/L-alanine.

### 3.2 Purification of OF4Ald, OF4DadX, and OF4Gdh recombinant proteins

**FIGURE 2:**
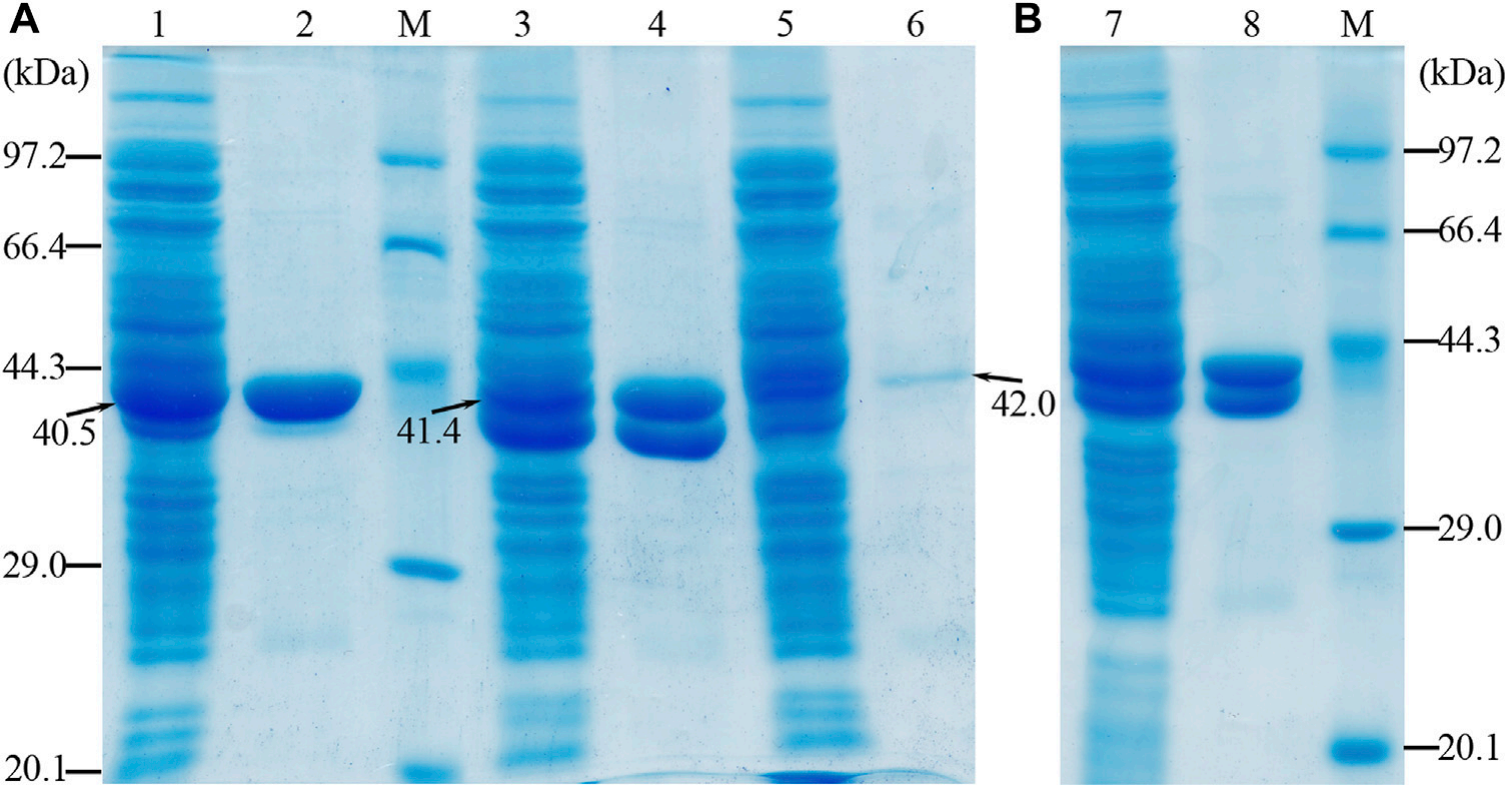
SDS-PAGE profile of purified protein OF4Ald, OF4DadX, and OF4Gdh. **(A)** Purification result of single gene expression **(B)** Purification result of co-expression of three genes. Lanes 1, 3, and 5: the crude enzyme solution of OF4Ald, OF4DadX, and OF4Gdh; lanes 2, 4, and 6: the purified protein OF4Ald, OF4DadX, and OF4Gdh, respectively; lanes 7&8: the crude enzyme solution of co-expression enzymes and purified proteins. M, protein marker (14.3, 20.1, 29.0, 44.3, 66.4, 97.2 kDa).

The apparent molecular masses of the purified proteins (OF4Ald, OF4DadX, and OF4Gdh) were detected about 40, 41, and 42 kDa by SDS-PAGE, which is in good agreement with the predicted molecular mass of 40.5, 41.4, and 42.0 kDa (Figure 2A). The purification analysis of individual gene expression revealed that purified proteins OF4Ald and OF4DadX showed a higher expression level than OF4Gdh (Figure 2A). Figure 2B shows the mixture of purified proteins from *E. coli* BL21(DE3) containing the co-expression plasmid pET-22bNS-Ald-DadX-Gdh. The molecular masses of three co-expressed proteins were close to each other and hard to distinguish individually (Figure 2B). In such a case, it is impossible to determine the expression level of each protein in the reaction mixture using SDS-PAGE.

### 3.3 Optimizing conditions for D/L-Alanine synthesis

#### 3.3.1 Optimal concentration of IPTG

**FIGURE 3:**
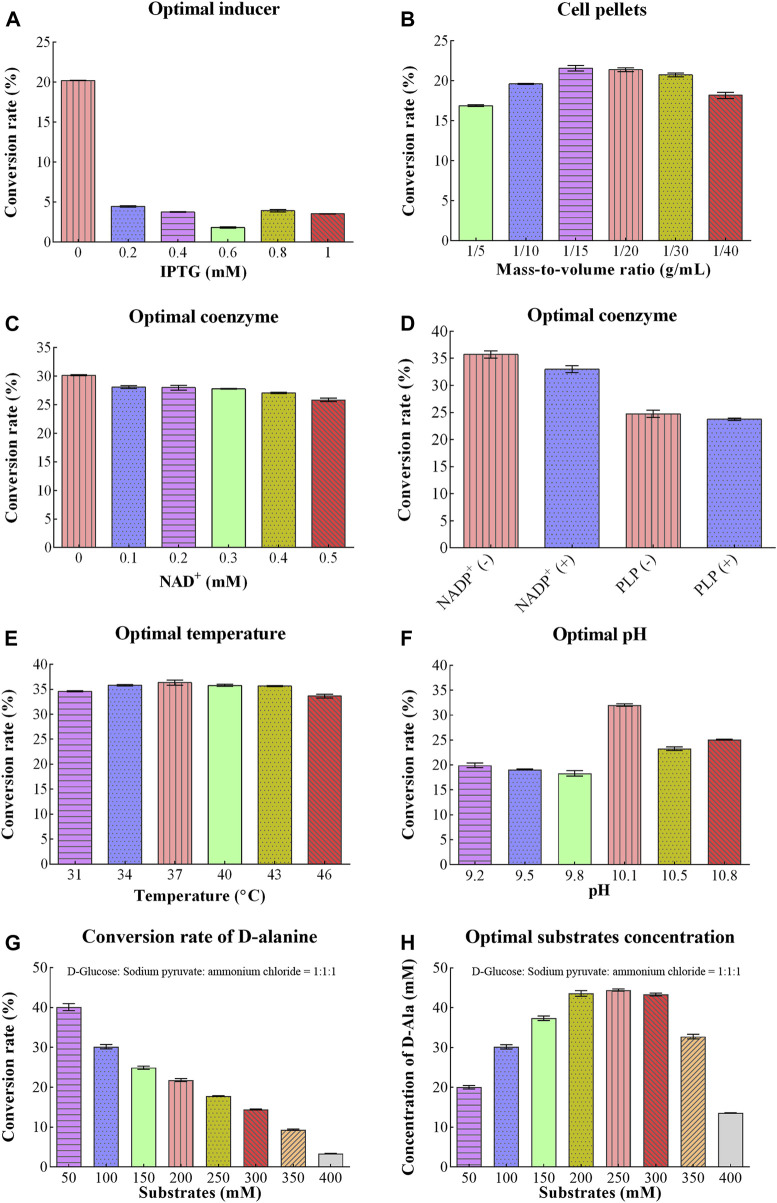
Optimizing conditions for D/L-Alanine synthesis: **(A)** Optimization of IPTG **(B)** Optimization of the mass-to-volume ratio of bacterial cell pellets **(C)** Effect of changing the concentration of NAD^+^coenzyme **(D)** Optimization of concentration of coenzymes NADP^+^and PLP **(E)** Optimization of temperature **(F)** Optimization of reaction pH **(G)** Conversion rate of D-alanine **(H)** Optimal substrates concentration. Error bars revealed the standard deviations.

Different concentrations of IPTG were optimized for D/L-alanine synthesis. Initially, when the concentration of IPTG solution was 0 mM, the conversion rate reached the maximum value of 20.19%. However, when concentration of IPTG was increased from 0.2 to 1.0 mM, the conversion rate sharply decreased to 1.83%–4.47% as shown in Figure 3A. The addition of IPTG might increase the production costs. Therefore, there is no need for the addition of an IPTG solution to achieve the maximum conversion rate of D/L-alanine.

#### 3.3.2 Optimal concentration of coenzymes

Concentrations of coenzymes were also optimized for D/L-alanine synthesis. Some coenzymes are produced in bacteria for normal growth. Therefore, they do not require exogenous coenzymes for the optimization process as their excessive concentration can cause damage to the cell membrane. Similarly, in our study, the conversion rate reached around 30.17% without NAD^+^ as shown in Figure 3C. However, as the concentration of NAD^+^ increased to 0.5 mM, the conversion rate gradually decreased to 25.84% as high concentration of NAD^+^ might interfere with bacterial metabolism. Similarly, the conversion rates were 35.71% ± 1.16% without NADP+, 32.98% ± 1.08% with NADP+, 24.75% ± 1.13% without PLP, and 23.74% ± 0.36% with PLP, respectively (Figure 3D). These results indicated that bacterial pellets containing the constructed plasmid was not require exogenous coenzymes NAD^+^ or NADP^+^ and PLP due to their endogenous production in bacteria. Since the addition of coenzymes might increase the production costs. Therefore, due to the perspective of experimental cost, there is no need for their addition for further optimization.

#### 3.3.3 Optimal temperature

From Figure 3E, the conversion rate of D/L-alanine showed about 33% during the temperature region (31°C–46°C). It increases slightly with increasing temperature (31°C–37°C), reaching a maximum value of about 36.37% at 37°C, and then decreases gently with a further increase in temperature (37°C–46°C). Thus, 37°C was selected as the optimal temperature to achieve a higher yield.

#### 3.3.4 Optimal pH

Optimum pH is another significant parameter that influenced the conversion rate of D/L-alanine. The maximum conversion rate of 32.03% was obtained with pH 10.1 of Na_2_CO_3_-NaHCO_3_ buffer (20 mM). However, the conversion rate showed a sharp decline when pH values were below or above 10.1. It was around 23.28%–25.10% at pH 10.5–10.8 and less than 20% in the pH range of 9.2–9.8, separately (Figure 3F). Notably, the reaction was optimized at pH 10.1 value for gaining the desired production rate.

#### 3.3.5 Optimal substrate concentration

D-glucose, pyruvate, and ammonium chloride were utilized as substrates for D/L-alanine biosynthesis. With 50 mM utilization of substates, the conversion rate reached about 40% (Figure 3G). Along with the increasing substrate concentration, the conversion rate declined sharply. It was only 3.4% when the substrate concentration reached about 400 mM. While the substrate concentration increased to 200 mM and 250 mM, the product concentration reached the maximum values (43.61 mM and 44.45 mM), respectively (Figure 3H). Considering the cost of the experiment, 200 mM was selected as the optimal substrate concentration for enhancing the production rate (Figure 3H).

#### 3.3.6 Mass-to-volume ratio of cell pellets

Changing the mass-to-volume ratio of bacterial cell pellets can affect the conversion rates of D/L-alanine. When the mass-to-volume ratio of cell pellets dropped to 1/15 g/mL and 1/20 g/mL, the conversion rate reached maximum levels of about 21.57% and 21.38% and eventually dropped down to 18% with a low ratio of 1/40 g/mL as shown in Figure 3B. Therefore, synthesis was carried out with 1/20 g/mL as the optimal mass-to-volume ratio of cell pellets.

#### 3.3.7 Effect of surfactants

**FIGURE 4:**
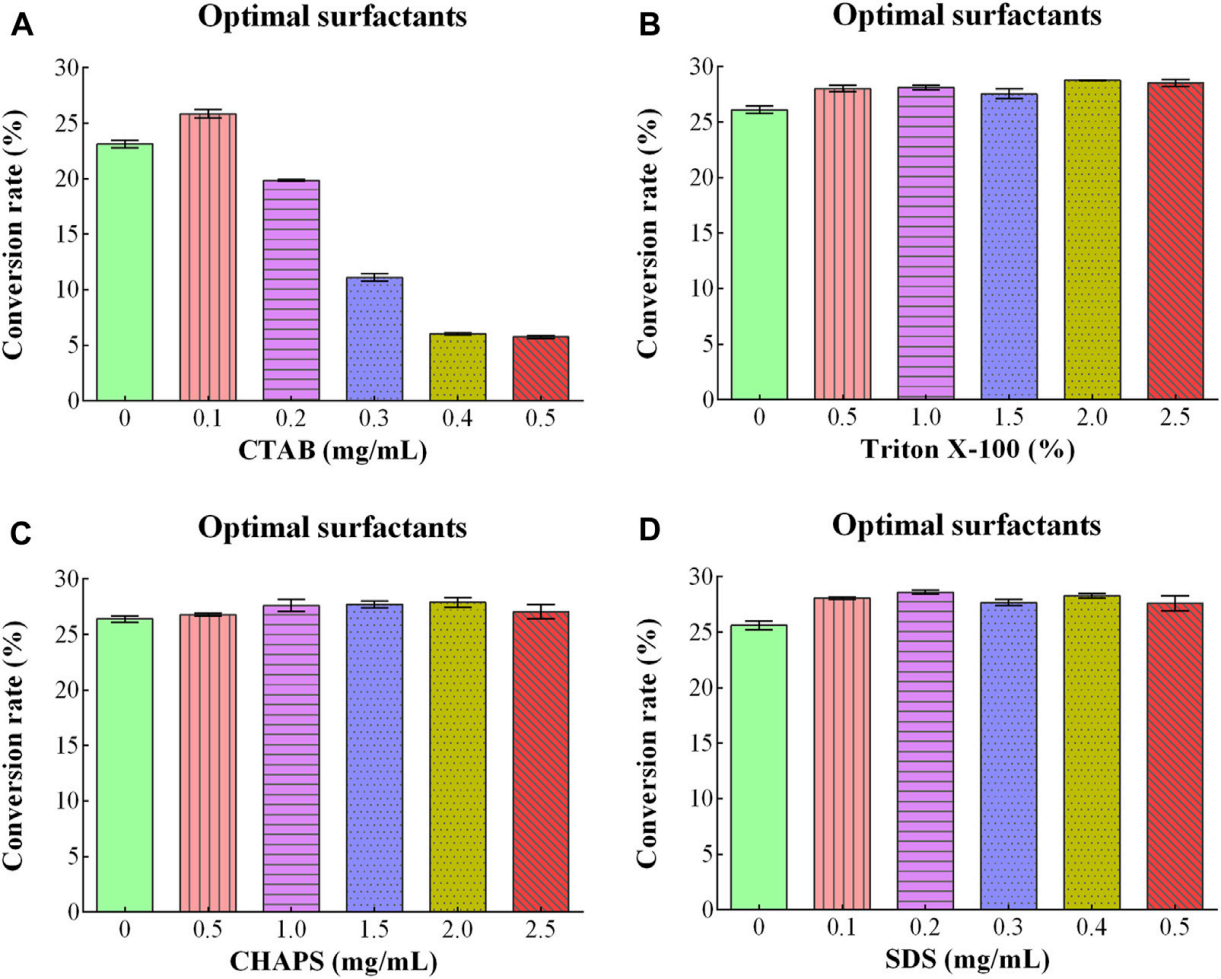
Optimizing conditions of different surfactants for D/L-Alanine synthesis: **(A)** Optimizing concentrations of CTAB **(B)** Triton X-100% **(C)** CHAPS **(D)** SDS. Error bars in the figures showed the standard deviations.

CTAB, Triton X-100, CHAPS, and SDS were used as surfactants, and their effect on D/L-alanine production was investigated. The high conversion rates of 23.13% and 25.85% were approached with the addition of 0.0 mg/mL and 0.1 mg/mL of CTAB. The high concentration (0.5 mg/mL) of CTAB ultimately decreased the conversion rate to 6% (Figure 4A). When Triton X-100, CHAPS and SDS were utilized, the conversion rates were between 26.11%-28.76%, 26.40%–27.89% and 25.62%–28.58%, respectively (Figures 4B–D). Since utilization of surfactants might increase the production costs. Therefore, they were not added to the reaction mixture due to the perspective cost of saving.

#### 3.3.8 Optimal reaction time

**FIGURE 5:**
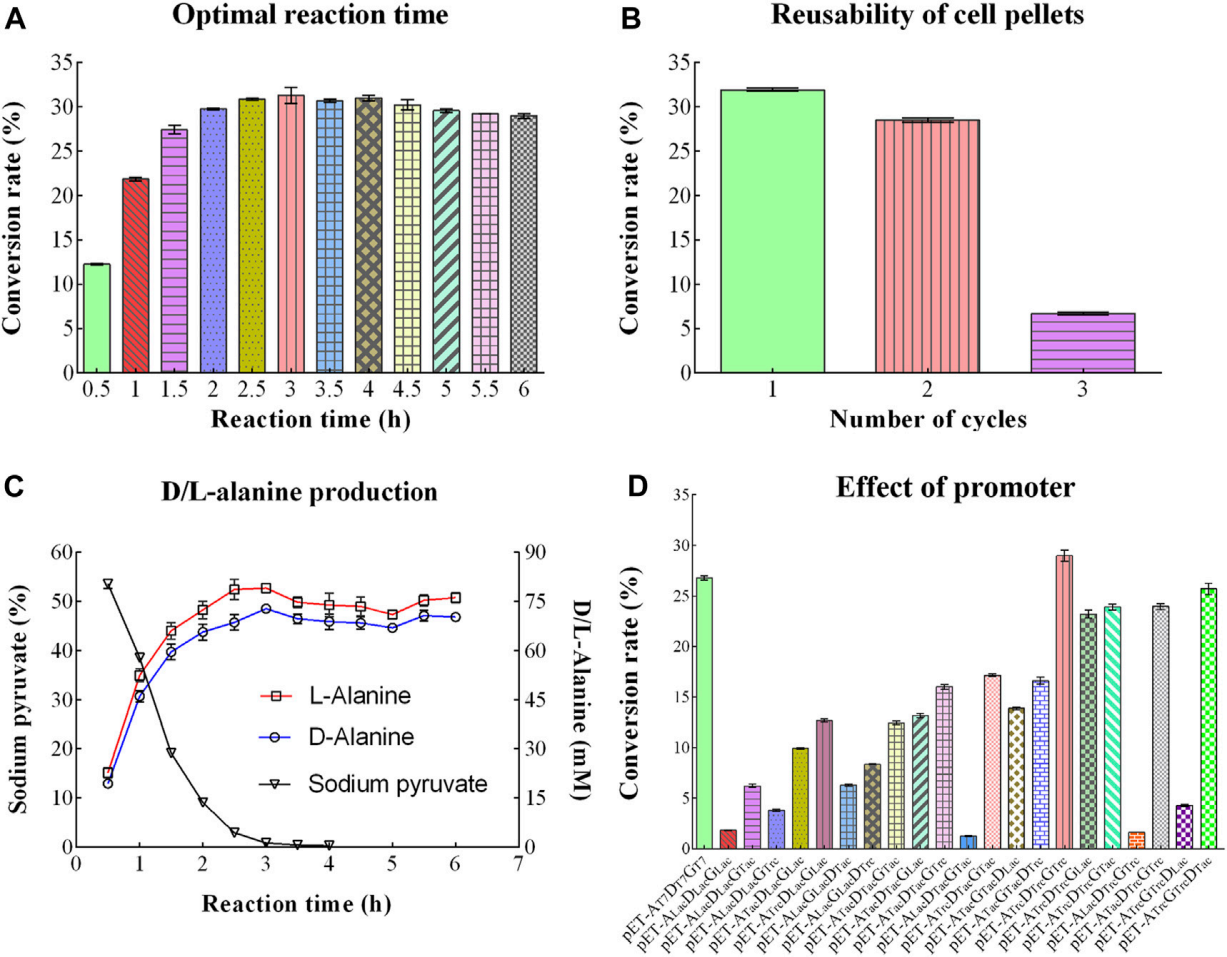
Other optimizing conditions for D/L-Alanine synthesis. **(A)** Optimal reaction time. **(B)** Reutilization times of cell pellets. **(C)** Changing trend of sodium pyruvate, D-Alanine, and L-Alanine production over time. **(D)** Effect of the promoter with different conversion rates. Error bars showed the standard deviations.

With the extension of reaction time (0.5–3.0 h), the conversion rate of D/L-alanine rapidly increased from 12.27% to the highest value of 31.32%. Then, the conversion rate slightly dropped down to 29.00% after 6 h incubation (Figure 5A). Therefore, 3.0 h incubation was selected as the optimal reaction time to achieve the high production rate.

#### 3.3.9 Reutilization times

To reduce the production cost, cell pellets were repeatedly used as the catalyst for D/L-alanine synthesis under the optimized reaction conditions. The conversion rate reached 31.94% and 28.51% after the first and second reactions, while it sharply decreased to 7.70% after the third reaction (Figure 5B). It indicates that enzymes in cell pellets might rapidly lose their activity after two rounds of reaction.

#### 3.3.10 D/L-Alanine production profile in reaction

To further investigate the trend of substrate consumption and product increases in the reaction system, sodium pyruvate, D-alanine, and L-alanine were detected using HPLC analysis. As shown in Figure 5C, with increasing reaction time, the concentration of sodium pyruvate dropped steeply and quickly reached 0.0% after a 3.0 h reaction. Meanwhile, the concentration of D/L-alanine increased rapidly with the extension of the reaction time and approached the maximum value of 79.08 mM (L-alanine, 7.05 mg/mL) and 72.79 mM (D-alanine, 6.48 mg/mL) at 3.0 h, respectively. The concentration of D/L-alanine was around 74.09 mM (L-alanine) and 69.13 mM (D-alanine) within 3.0–6.0 h.

#### 3.3.11 Effect of promoter

To further increase the conversion rate of D/L-alanine, 21 co-expression plasmids with different promoter combinations were constructed. However, only the recombinant strain with plasmid pET-22bNS-Ald_Trc_-DadX_Trc_-Gdh_Trc_ showed a slightly higher conversion rate (28.97%) than the strain containing plasmid pET-22bNS-Ald-DadX-Gdh (26.79%) (Figure 5D). The conversion rate of three bacteria containing plasmids (pET-22bNS-Ald_Lac_-DadX_Lac_-Gdh_Lac_, pET-22bNS-Ald_Lac_-DadX_Tac_-Gdh_Tac_, pET-22bNS-Ald_Lac_-DadX_Trc_-Gdh_Trc_) was close to the lowest value (1.82%, 1.25% and 1.62%), respectively.

**FIGURE 6:**
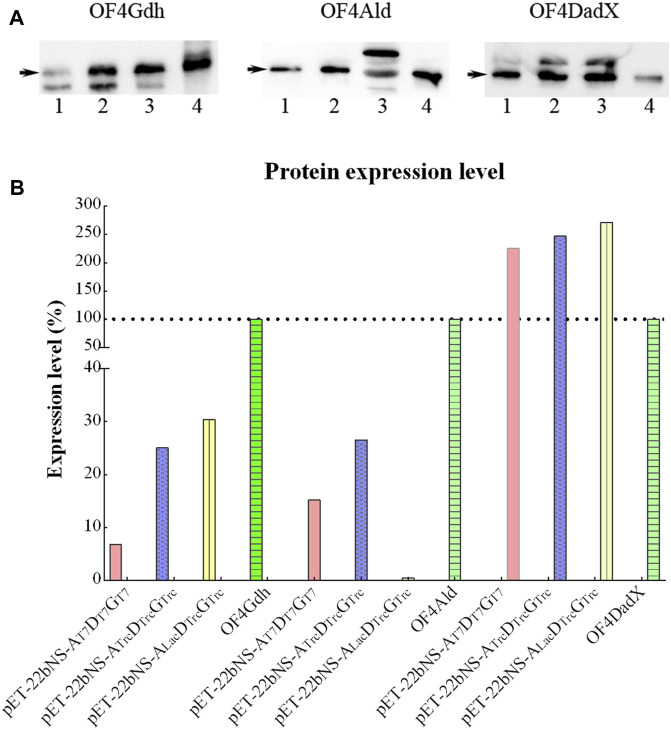
Expression level of different proteins. **(A)** Expression level of OF4Ald, OF4DadX, and OF4Gdh through Western blot analysis. **(B)** Comparison of expression levels of different proteins relative to the corresponding purified protein (set as 100%).

To evaluate the possible cause of low conversion rate, expression levels of three proteins OF4Ald, OF4DadX, and OF4Gdh were explored through Western blot analysis (Figure 6A). In consideration of the different dilutions of each protein in the Western bolt experiment, the expression level of each protein relative to the corresponding purified protein (set as 100%) was calculated and analyzed using Image Lab software (Bio-Rad, Hercules, CA, United States) (Figure 6B). In comparison with three purified proteins, only protein OF4DadX from the recombinant strain showed much higher expression level than the purified protein OF4DadX (100%), the relative expression levels of OF4DadX from plasmids pET-22bNS-Ald-DadX-Gdh(pET-A_T7_D_T7_G_T7_),pET-22bNS-Ald_Trc_-DadX_Trc_-Gdh_Trc_ (pET-A_Trc_D_Trc_G_Trc_) and pET-22bNS-Ald_Lac_-DadX_Trc_-Gdh_Trc_ (pET-A_Lac_D_Trc_G_Trc_) were 225.21%, 247.12%, and 271.58%, respectively. However, the relative expression level of protein OF4Ald of recombinant strain containing plasmid pET-22bNS-Ald_Lac_-DadX_Trc_-Gdh_Trc_ dropped almost to a desperate value (0.42%), which explains the reason for the low conversion rate of that recombinant strain (Figure 5D, 1.62%), indicating that the expression level of protein OF4Ald had a significant effect on the conversion rate of D/L-alanine.

## 4 Discussions

Alanine is a main constituent of different cellular proteins essential for the human body. It is widely used in the biomedical, plastics, and food industries with annual demand has reached more than 50,000 tons in the global market (Li et al., 2018). Currently, D/L-alanine biosynthesis can be carried out through chemical, fermentation, coupling, and biocatalytic methods. However, the above methods are not suitable for high-scale D/L-alanine synthesis due to some limitations such as complicated assembly process, genetic manipulation of larger genetic circuits, additional cost of cloning operations, and low expression (Fan et al., 2021). To overcome these limitations, we employed the biobrick method for efficient D/L-alanine biosynthesis. This method has several advantages over conventional methods. Firstly, the design of biobrick assembly is simple and more reliable for the manipulation of larger genetic circuits (Yamazaki et al., 2017). On the other hand, the design of fermentation or coupling methods is much more complicated for manipulation of larger genetic circuits. In contrast to traditional methods, several genes can be assembled by biobrick method into the recombinant expression plasmid rather than single gene transfer thereby facilitating the high expression level of several genes. Moreover, biobrick methods are cost-effective because there is no need for additional cloning which reduces the experimental costs (Braman and Sheffield, 2019). On the other hand, additional cloning is required in the traditional cloning methods which increases the experimental costs (Chaudhari and Hanson, 2021). Due to these features, biobrick method has emerged as an efficient approach for designing novel vectors that could be employed for biosynthesis of the novel products.

The high amount of D/L-alanine was achieved in our findings by biobrick method and was compared with previously reported studies. D/L-alanine can be produced by different methods (Gu et al., 2023). Through fed-batch fermentation method, *E. coli* strain expressed the alanine dehydrogenase (Ald) from *Arthrobacter* spp. and produced only 8.1 g/L of D/L-alanine in 40 h (Katsumata and Hashimoto, 1996). Smith et al. (2006) employed the biocatalytic method and investigated that *E. coli* mutant (*pfl*/*pps*/*pox*B/*ldh*A/*ace*EF) expressed the alanine dehydrogenase (Ald) from *Bacillus sphaericus* and produced only 88 g/L of D/L-alanine in 48 h. Zhang et al. (2023) utilized the coupling method and found that *Bacillus licheniformis* had produced 12 g/L of D/L-alanine with additional formation of poly-γ-glutamic acid. However, productivity through these methods is still low due to some limitations such as difficulty in maintaining the optimizing conditions throughout the process greatly impact the product quality and produces low yield (Zhang et al., 2023). Therefore, these methods could not be operated for high-scale production. To overcome these limitations, we employed the biobrick method in which three genes, alanine racemase (*dadX*_OF4_), alanine dehydrogenase (*ald*_OF4_), and glucose dehydrogenase (*gdh*_OF4_) were cloned from *B. pseudofirmus* OF4 for the construction of co-expression plasmid pET-Ald-DadX-Gdh. Under optimal reaction conditions, the amounts of D-alanine and L-alanine were approached around 6.48 g/L and 7.05 g/L after 3.0 h. The theoretical synthetic efficiencies of D-alanine and L-alanine reached about 51.88 g/L/d and 56.36 g/L/d, which was higher than previously reported studies (Zhang et al., 2007; Zhou et al., 2016; Han et al., 2023). These results indicated that co-expression plasmid pET-Ald-DadX-Gdh through this approach had high efficiency for D/L-alanine biosynthesis.

The protein expression level is another important factor affecting the efficiency of D/L-alanine biosynthesis (Miyamoto and Homma, 2021). The expression level of each recombinant protein is regulated by different promoters (Zhang Z.-X. et al., 2022). Promoter replacement experiments demonstrated that except for the plasmid pET-22bNS-Ald_Trc_-DadX_Trc_-Gdh_Trc_ (28.97%), the conversion rate of all other recombinant strains was lower than pET-22bNS-Ald-DadX-Gdh (26.79%). Western blot analysis revealed that the expression level of OF4Ald protein had a significant effect on the synthetic efficiency of D/L-alanine (Figure 5D). Therefore, OF4Ald might be a rate-limiting enzyme in the D/L-alanine biosynthesis (Gu et al., 2023). Screening of alanine dehydrogenases with high expression level could be useful for improving the efficiency of D/L-alanine biosynthesis (Tran and Brown, 2022).

Our findings are consistent with the previous studies. Different conditions were optimized in the whole-cell catalytic reaction to improve the synthetic efficiency of D/L-alanine (Xin et al., 2022). No IPTG was optimal as shown in Figure 3A, because leaky expression of proteins normally happens in *E. coli* cells. It is reported that the leaky promoter system that can take advantage for the expression of recombinant proteins without induction of IPTG. This system allows the high-level production and expression of the recombinant proteins in the absence of IPTG (Zhang F. et al., 2023; Briand et al., 2016). Moreover, it is reported that lactose also present in the medium assisted for expression of proteins. A recent study by Zhang et al. (2007) showed that an adequate supply of sodium pyruvate in the whole cell catalysis promotes the D/L-alanine biosynthesis. Our findings are consistent with the previous studies that sodium pyruvate was added to the reaction mixture because it carried out the reduction amination of pyruvate. When the concentration of sodium pyruvate was optimized, the product concentrations of D-alanine and L-alanine were also improved (Zhang et al., 2007). Another study by He et al. (2018) reported that the pH of the reaction was optimized around 10.0 for efficient D/L-alanine biosynthesis. Similarly, in our findings, the pH of the reaction was optimized around 10.5 in the whole cell reaction which promotes the D/L-alanine biosynthesis and agreed with the previous studies (He et al., 2018). Another study by Nie et al. (2024) showed that whole-cell catalysis at 37°C accelerates the D/L-alanine biosynthesis in the *E. coli* W3110 strain. Similarly, in our study, the reaction temperature was optimized at 37°C which stimulates the D/L-alanine biosynthesis. Moreover, biosynthesis was further enhanced through utilizing the 1/20 g/mL concentration of bacterial cell pellets (Zhang et al., 2007; Gmelch et al., 2019). These optimizing reaction conditions could favor the efficient biosynthesis of D/L-alanine.

Alanine racemase catalyzed the racemization reaction by converting the L-alanine into D-alanine with *K*_eq_≈1.0 value (Strych et al., 2007). Dong et al. (2018) purified the alanine racemase OF4DadX from *B. pseudofirmus* and *K*_eq(L/D)_ of the racemization reaction reached around 1.01. Therefore, the ratios of D-alanine and L-alanine in the reaction mixture were close to 1:1 (6.48 g/L and 7.05 g/L). Due to their similar physical and chemical properties, it is difficult to obtain the pure product of D-alanine (Dong et al., 2018). Israr et al. (2019) constructed a double-point mutant D171A/Y359H by site-directed mutagenesis with *K*_eq(L/D)_ value of around 2.6. Their research team constructed another mutant by saturated mutagenesis with a high *K*_eq(L/D)_ value around 3.6, and the reaction was accelerated by optimizing the D-alanine concentration which was 20% higher than the wild type (Unpublished data). It was suggested that alanine racemase variants obtained through directional evolution or screening of novel alanine racemases from different species with high catalytic activity could be an effective approach for solving this bottleneck.

## 5 Conclusion

In this study, biobrick method was explored for efficient production of D/L-alanine utilizing the *E. coli* strain BL21(DE3) as the microbial cell factory. The expression level of the *ald* gene was significantly adjusted by the regulation of the promoter, which affected the production of D/L-alanine. Under optimal conditions, the high amount of D-alanine and L-alanine in the final titer (6.48 g/L and 7.05 g/L) was achieved in the whole cell catalytic reaction after 3.0 h. Therefore, our study might provide a simple and promising approach for the efficient synthesis of D/L-alanine.

## Data Availability

The datasets presented in this study can be found in online repositories. The names of the repository/repositories and accession number(s) can be found in the article/Supplementary Material.
